# Supplementary figures and images for: Long term absence of invasive breast cancer diagnosis in 2,402,672 pre and postmenopausal women: A systematic review and meta-analysis
Source: PLoS One. 2020 Sep 10;15(9):e0237925. doi: 10.1371/journal.pone.0237925 (PMC7482842; doi:10.1371/journal.pone.0237925)

S2 Table Steps 1, 2 and 3 of the First Records Search


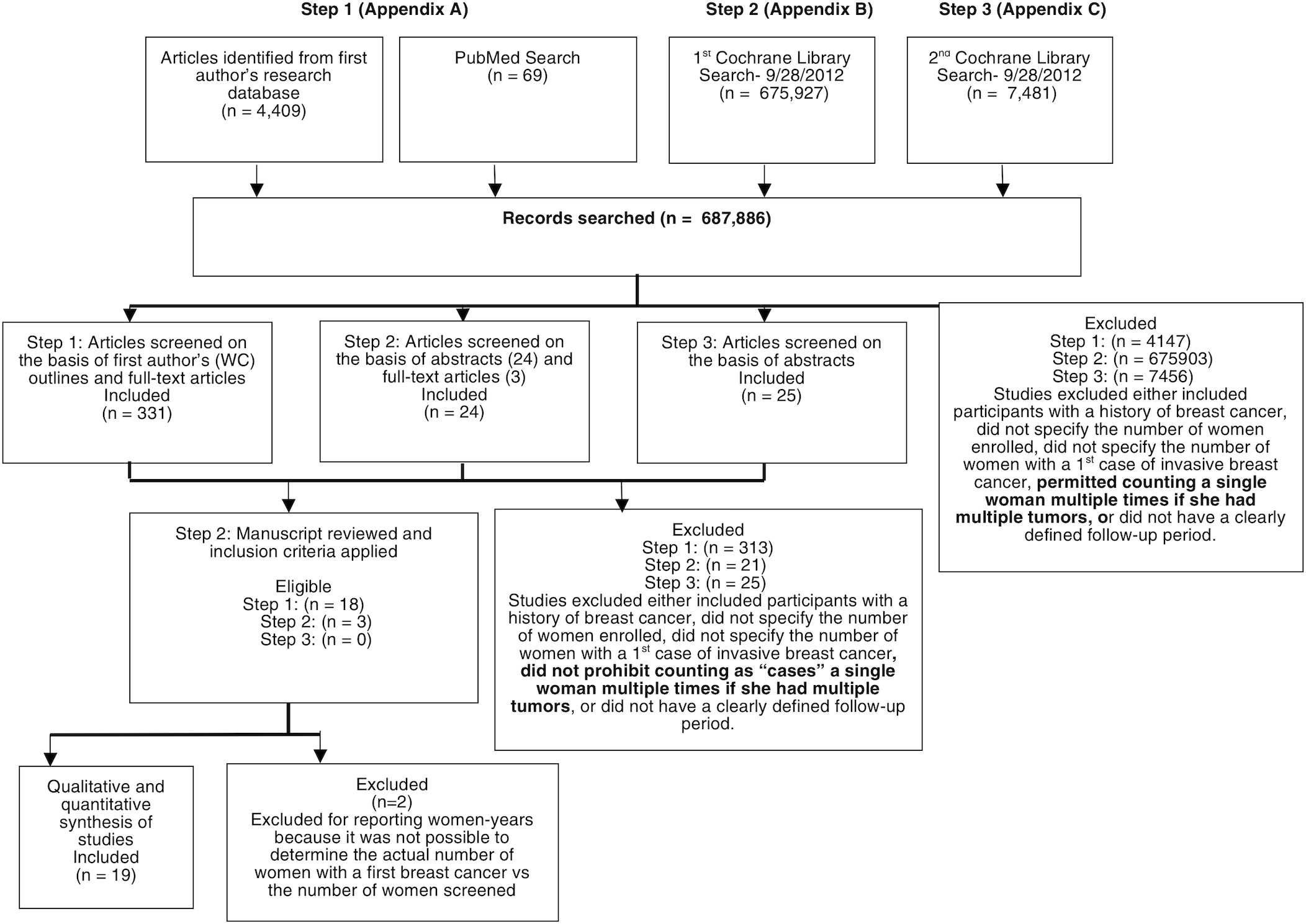

Supplement: S2 Table — (DOCX) [file pone.0237925.s002.docx]
