# Supplementary material for: Long term absence of invasive breast cancer diagnosis in 2,402,672 pre and postmenopausal women: A systematic review and meta-analysis
Source: PLoS One. 2020 Sep 10;15(9):e0237925. doi: 10.1371/journal.pone.0237925 (PMC7482842; doi:10.1371/journal.pone.0237925)
Supplement: S3 Table — (DOCX) [file pone.0237925.s003.docx]

*Step Two: 1^st^ Cochrane Library Search- 4/10/19

(n= 54)

*Step Three: 2^nd^ Cochrane Library Search- 4/10/19

(n= 3,392)

Records searched (n=18,033)

Step One: Articles screened on the basis of titles and abstracts

(n=17)

Step Two: Articles screened on the basis of titles and abstracts

(n= 5)

Step Three: Articles screened on the basis of titles and abstracts

(n=11)

Excluded:

Step 1: (n= 14,570)

Step 2: (n= 49)

Step 3: (n= 3,381)

Studies excluded either included participants with a history of breast cancer, did not specify the number of women involved, did not specify the number of women with 1^st^ case invasive breast cancer, **permitted counting a woman multiple times if she had multiple tumors,** or did not have clearly defined follow-up period

Full-text manuscripts reviewed and inclusion criteria applied

Eligible: Step one: (n=2)

Step two: (n=1)

Step three: (n=0)

Qualitative and quantitative analysis of studies included

(n=3)

Excluded:

Step 1: (n=15)

Step 2: (n=4)

Step 3: (n=11)

Studies excluded either included participants with a history of breast cancer, did not specify the number of women involved, did not specify the number of women with 1^st^ case invasive breast cancer, **permitted counting a woman multiple times if she had multiple tumors,** or did not have clearly defined follow-up period

*Step One: PubMed Search -4/10/19

(n= 14,587)

*Search limited to publications between September 2012 and April 2019
