# Supplementary material for: Long term absence of invasive breast cancer diagnosis in 2,402,672 pre and postmenopausal women: A systematic review and meta-analysis
Source: PLoS One. 2020 Sep 10;15(9):e0237925. doi: 10.1371/journal.pone.0237925 (PMC7482842; doi:10.1371/journal.pone.0237925)
Supplement: S4 Table — Extended sensitivity analysis using binary variables. (DOCX) [file pone.0237925.s004.docx]

**S4 Table Supplement 1 to Table 4 – Extended Sensitivity analysis**

***Panel A: Generalized Least Squares (GLS) results***

*Dependent Variable: Percentage of women* ***without*** *invasive breast cancer (IBC), i.e., the “survival rate”, at the end of each study.*

|  | (1) | (2) | (3) | (4) | (5) |
| --- | --- | --- | --- | --- | --- |
|  | Full  sample  of all studies | Subsample  of studies limited to post-menopausal women | Subsample  of studies limited to  screened  women | Full  sample  of all  studies | Full  sample  of all  studies |
| Effect of each | **-0.255***** | -0.228*** | -0.256*** | -0.246*** | -0.255*** |
| additional year of follow-up | **(0.0000)** | (0.0000) | (0.0000) | (0.0000) | (0.0000) |
|  |  |  |  |  |  |
| Post- |  |  |  | 0.257 |  |
| menopausal women binary variable |  |  |  | (0.2250) |  |
|  |  |  |  |  |  |
| Screened women |  |  |  |  | 0.0854 |
| binary variable |  |  |  |  | (0.8151) |
|  |  |  |  |  |  |
| Constant | 99.78*** | 99.71*** | 99.79*** | 99.54*** | 99.70*** |
|  | (0.0000) | (0.0000) | (0.0000) | (0.0000) | (0.0000) |
| N (Outcomes) | 24 | 16 | 17 | 24 | 24 |
| N (Studies) | 21 | 14 | 14 | 21 | 21 |
| N (Women) | 2,402,672 | 1,808,022 | 2,264,591 | 2,402,672 | 2,402,672 |
| R-squared | 0.920 | 0.984 | 0.921 | 0.926 | 0.920 |

Statistical *p*-values are in parentheses (^**^ *p* < 0.01, ^***^ *p* < 0.001).

The generalized least squares (GLS) regression method corrects the heteroskedasticity induced by varying number of outcomes across studies, and therefore heterogeneous variance and heterogeneous precision in our regression models.

Column 1 shows that each additional year of follow-up is associated with an average decline in the “survival rate” of about one-fourth of one percent (0.255 percentage points) per year, and this result is obtained using the full sample of 24 outcomes stemming from the 21 studies included in this meta-analysis, which span 2,402,672 women. Column 2 shows similar results for the subsample of postmenopausal women (N=16 outcomes, 14 studies, 1,808,022 women). Column 3 shows similar results for the subsample of screened women (N=17 outcomes, 14 studies, 2,264,591 women).

To test the impact of different subgroups on the findings, a binary-variable statistical test is conducted. Columns 4 and 5 use the full sample (as in column 1) but add binary variables to tag the studies that included only post-menopausal and only screened women, respectively. In columns 4 and 5, we see that our binary variables were not statistically significant (*p*>0.05). This finding confirms that there are no significant differences in the “survival rate” between the two important subgroup of interest (post-menopausal) and the rest of the sample, and screened women and the rest of the sample. The large *p*-values (no significant difference) for these two binary variables (0.225 and 0.8151) suggest that the differences in slope between the subgroups and the full sample are essentially due to random variation in the data. This result implies that there is no statistical evidence that the slopes of columns 1, 2, and 3 (corresponding to the slopes of figures 1, 2, and 3) are significantly different from each other.

***Panel B: Ordinary Least Squares (OLS) results***

*Dependent Variable: Percentage of women* ***without*** *invasive breast cancer (IBC), i.e., the “survival rate”, at the end of each study.*

|  | (1) | (2) | (3) | (4) | (5) |
| --- | --- | --- | --- | --- | --- |
|  | Full  sample  of all studies | Subsample  of studies limited to post-menopausal women | Subsample  of studies limited to  screened  women | Full  sample  of all  studies | Full  sample  of all  studies |
| Effect of each | **-0.267^***^** | -0.235^***^ | -0.268^***^ | -0.263^***^ | -0.265^***^ |
| additional year of follow-up | **(0.0000)** | (0.0000) | (0.0000) | (0.0000) | (0.0000) |
|  |  |  |  |  |  |
| Post- |  |  |  | 0.0861 |  |
| menopausal women binary variable |  |  |  | (0.7858) |  |
|  |  |  |  |  |  |
| Screened women |  |  |  |  | -0.0789 |
| binary variable |  |  |  |  | (0.7831) |
|  |  |  |  |  |  |
| Constant | 99.92^***^ | 99.74^***^ | 99.91^***^ | 99.83^***^ | 99.96^***^ |
|  | (0.0000) | (0.0000) | (0.0000) | (0.0000) | (0.0000) |
| N (Outcomes) | 24 | 16 | 17 | 24 | 24 |
| N (Studies) | 21 | 14 | 14 | 21 | 21 |
| N (Women) | 2,402,672 | 1,808,022 | 2,264,591 | 2,402,672 | 2,402,672 |
| R-squared | 0.891 | 0.852 | 0.889 | 0.892 | 0.892 |

Statistical *p*-values are in parentheses (^**^ *p* < 0.01, ^***^ *p* < 0.001).

Column 1 shows that each additional year of follow-up is associated with an average decline in the “survival rate” of about one-fourth of one percent (0.267 percentage points) per year, and this result is obtained using the full sample of 24 outcomes stemming from the 21 studies included in this meta-analysis, which span 2,402,672 women. Column 2 shows similar results for the subsample of postmenopausal women (N=16 outcomes, 14 studies, 1,808,022 women). Column 3 shows similar results for the subsample of screened women (N=17 outcomes, 14 studies, 2,264,591 women).

To test the impact of different subgroups on the findings, a binary-variable statistical test is conducted. Columns 4 and 5 use the full sample (as in column 1) but add binary variables to tag the studies that included only post-menopausal and only screened women, respectively. In columns 4 and 5, we see that our binary variables were not statistically significant (*p*>0.05). This finding confirms that there are no significant differences in the “survival rate” between the two important subgroup of interest (post-menopausal) and the rest of the sample, and screened women and the rest of the sample. The large *p*-values (no significant difference) for these two binary variables (0.7858 and 0.7831) suggest that the differences in slope between the subgroups and the full sample are essentially due to random variation in the data. This result implies that there is no statistical evidence that the slopes of columns 1, 2, and 3 (corresponding to the slopes of figures 1, 2, and 3) are significantly different from each other.
