# Supplementary material for: Long term absence of invasive breast cancer diagnosis in 2,402,672 pre and postmenopausal women: A systematic review and meta-analysis
Source: PLoS One. 2020 Sep 10;15(9):e0237925. doi: 10.1371/journal.pone.0237925 (PMC7482842; doi:10.1371/journal.pone.0237925)
Supplement: S5 Table — Regression analysis excluding the UK Million Women Study. (DOCX) [file pone.0237925.s005.docx]

**S5 Supplement 2 to Table 4 – Regression analysis excluding the UK Million Women Study**

*Dependent Variable: Percentage of women* ***without*** *invasive breast cancer (IBC), i.e., the “survival rate”, at the end of each study.*

|  | (1) | (2) | (3) | (4) | (5) |
| --- | --- | --- | --- | --- | --- |
|  | Full  sample  of all studies | Subsample  of studies limited to post-menopausal women | Subsample  of studies not limited to menopausal women | Subsample  of studies limited to  screened  women | Subsample  of studies not limited to screened women |
| Effect of each | -0.252*** | -0.209*** | -0.254*** | -0.253*** | -0.214** |
| additional year of follow-up | (0.0000) | (0.0000) | (0.0005) | (0.0000) | (0.0026) |
|  |  |  |  |  |  |
| Constant | 99.74*** | 99.54*** | 99.61*** | 99.76*** | 99.52*** |
|  | (0.0000) | (0.0000) | (0.0000) | (0.0000) | (0.0000) |
| N (Outcomes) | 23 | 15 | 8 | 16 | 7 |
| N (Studies) | 20 | 13 | 7 | 13 | 7 |
| N (Women) | 1,318,562 | 723,912 | 594,650 | 1,180,481 | 138,081 |
| R-squared | 0.885 | 0.960 | 0.882 | 0.880 | 0.861 |

Statistical *p*-values are shown in parentheses (^**^ *p* < 0.01, ^***^ *p* < 0.001). The generalized least squares (GLS) regression method was used to correct for heteroskedasticity stemming from unequal error variances across the disparate studies.

Column 1 shows that each additional year of follow-up for women in studies other than the UK Million Women Study is associated with an average decline in the “survival rate” of one-fourth of one percentage point (0.252 percentage points) per year. Expressed in non-technical language; about ¼ of 1% of women per year lose their freedom from a diagnosis of IBC or 99 ¾% retain their freedom each year.

Columns 2 and 3 show similar results for the subsamples of studies including only postmenopausal women (N=15 outcomes, 13 studies, 723,912 women) and studies not limited to menopausal women (N=8 outcomes, 7 studies, 594,650 women), respectively. Columns 4 and 5 show similar results for the subsamples of studies including only screened women (N=16 outcomes, 13 studies, 1,180,481 women) and studies not necessarily limited to screened women (N=7 outcomes, 7 studies, 138,081 women).

The flattest lines, *i.e.*, the least decline per year, correspond to the subsamples of post-menopausal women (column 2) and studies that included non-screened women (column 5). The coefficient of -0.209 in column 2 indicates that one additional year of follow-up for post-menopausal women not in the UK Million Women Study is associated with an average decline in the “survival rate” of 0.209 percentage points per year, *i.e.*, about one-fifth of one percent decline per year.
