# Supplementary material for: Long term absence of invasive breast cancer diagnosis in 2,402,672 pre and postmenopausal women: A systematic review and meta-analysis
Source: PLoS One. 2020 Sep 10;15(9):e0237925. doi: 10.1371/journal.pone.0237925 (PMC7482842; doi:10.1371/journal.pone.0237925)
Supplement: S1 Text — (DOCX) [file pone.0237925.s006.docx]

S1 Text. Detailed methods of successive literature searches

We examined a unique index of published papers in the field of reproductive and behavioral endocrinology of aging women compiled by the first author, Winnifred Cutler (WC) from 1974–2008 for 7 textbooks she ^[[1]](#endnote-1)^ (S2 Table). The index consisted of menopause-related papers that had been reviewed, outlined and indexed. Importantly, the index included methodology and findings about breast cancer diagnoses in papers primarily focused on other topics.

The cross-indexing system enabled identification of the papers pertinent to the current study. Outlines of these studies were reviewed, to evaluate whether the study met inclusion criteria. When necessary, the full-text publications were retrieved for closer examination. i (S1 Text; S1 File; S2 File; S2 Text).

Article identification continued (Appendix 1, Step 1) with a PubMed search for references using the search terms "breast cancer and menopause or hormone therapy (or HRT)" for articles published from 2008 through June 2012 (Appendix 1, S2 Table). Dr. Regula Burki and Dr. James Kolter, coauthors of that work, scanned their medical society notices also contributing any references identified by their routine medical education reports over the 2-year active phase of article identification. These results were combined with the outlines extracted from WC’s database.i

At least one coauthor and two independent readers (Debbie McLaughlin [DM], Emily Short [ES]) reviewed every study that reported findings on breast cancer incidence. WC assessed eligibility, based on the five inclusion criteria. DM and ES also reviewed the selections. Disagreements were resolved by consensus.

Two searches of the Cochrane Library databases (Appendix 1, Steps 2 and 3) subsequently identified all eligible screening trial articles. For the first search we used the phrase, “Breast cancer incidence during screening,” (S2 Table), and for the second search we used the phrase, “Mammography or hormone use and breast cancer” (S2 Table). Both searches were current to September 28, 2012. Finally, citations in the papers that had been identified were examined to discover additional articles to evaluate; these did not return additional studies.

No language or study design restrictions were imposed on any of the searches. When multiple publications referenced the same study, we included the latest publication that reported on the complete enrolled sample of women. Three readers (WC, DM, and ES) reviewed potentially eligible studies and excluded those that did not meet the criteria.

After determining potential eligibility, the three readers extracted information from eligible studies: number of women enrolled; number of women diagnosed with a first invasive breast cancer; length of follow-up; and, characteristics of trial participants and inclusion/exclusion criteria (including age at enrollment, cancer history, surgical history, health habits, and environmental exposures). For six studies, we contacted the original investigators to verify that their study met our inclusion criteria and the counts we extracted were accurate.

The current study expanded the original data set and search terms to include data from all relevant new screening studies uncovered in an exhaustive search of 18,083 new records. This was conducted by Research Intern Paige Szmodis. Two coauthors (WC and JK) reviewed 23 potentially eligible studies as described for the first search above; and shown in the S3 Table.

This updated search revealed one study (see Table 2, Study 19), that had been extended from 7 years, to 22 years duration and 2 new studies meeting all inclusion criteria. The senior author of that Canadian 25-year study confirmed to WC that the new detailed findings presented in Tables 2 and 3 of the present paper were accurately represented. S2 Table and S3 Table provide further specific details of each search.

1. Cutler W, Bürki R, Kolter J, Chambliss C, Friedmann E, Hart K. Invasive Breast Cancer Incidence in 2,305,427 Screened Asymptomatic Women: Estimated Long Term Outcomes during Menopause Using a Systematic Review. **PLOSone** 2015;10(6): e0128895. [↑](#endnote-ref-1)
