# Supplementary material for: Long term absence of invasive breast cancer diagnosis in 2,402,672 pre and postmenopausal women: A systematic review and meta-analysis
Source: PLoS One. 2020 Sep 10;15(9):e0237925. doi: 10.1371/journal.pone.0237925 (PMC7482842; doi:10.1371/journal.pone.0237925)
Supplement: Attachment — Submitted filename: Response to Reviewers.docx [file pone.0237925.s007.docx]

**5. Review Comments to the Author**

Please use the space provided to explain your answers to the questions above. You may also include additional comments for the author, including concerns about dual publication, research ethics, or publication ethics. (Please upload your review as an attachment if it exceeds 20,000 characters)

**Reviewer #1**: This manuscript that provides an alternative to breast cancer risk using cohort studies across the globe makes an intriguing assertion. Specifically, the calculation used to assess breast cancer risk, and therefore recommendations for mammogram frequency though cancer registry data is flawed for menopausal women. Overall, this is a great paper but needs some edits/analyses to further strengthen this. It does provide a strong and important message.

Some specific comments are below
1.

Line 81. I don’t understand how many of the participants enrolled at younger ages (age 39, 35, 40, etc) are considered perimenopause without there being clear indication in the manuscripts that women are experiencing hormonal changes. Given the average age is early 50s and symptoms associated with hormonal changes averages 6-8 years, many of the younger women are likely not in the perimenopausal phase. Unless this was determined, then the inclusion is just women with no IBC hx at enrollment would be more accurate. Recommend not using the perimenopausal to partially describe this population.

**………………**

**Response:**

Your questioning how participants of ages in their mid 30’s etc. could be considered “perimenopausal” is certainly understandable but is explained by our classifying them as “surgically menopausal at enrollment.”  Two studies you pointed out #11 and #14, with age ranges of 35-84 and 35-65 respectively, *were in the postmenopausal group because of bilateral ovariectomy.*

We agree that clarification is needed and have made several changes

1.1 **We have added a new graphic:**

**“Figure 2: Percentage of Post-Menopausal Women Sill Free From IBC Diagnosis Relative to the Duration of Clinical Study (Years of Follow-Up) in 14 Studies (16 Observations) Comprising 1,808,022 Women**”.

This subgroup was already described in the paragraphs beginning with the original line 206, now line 219… but the new Figure should help focus attention on the similar pattern in their data to the overall pattern of all 21 studies. Altogether, 14 of the 21 studies met the criteria: each of these 14 included only women who were at least 50 years old or surgically menopausal at enrollment.

1.2 **The Legend below the graphic identifies the 14 studies that included only women who were at least 50 years old or surgically menopausal at enrollment.**

As to the remaining 7 studies that involved peri and postmenopausal women: only one study [#9 ] enrolled any women under 40. The study examined the entire female population of the city of Gothenburg, Sweden, born between 1923 and 1944, inclusive. The women ranged from 39 to 59 at inception with 14 years of follow up. We decided it qualified as a study of peri/post menopause because it met the criteria of investigating a cohort of women in their peri and postmenopausal years.

For the 3 studies that enrolled women who were at least 40:#15,18, and 19 we also looked closely at each before qualifying them as studying women in their peri/postmenopausal era, since the term “perimenopausal” is a loose one – but does convey our intent. All the women were studied prospectively. Each limited their participants to an age range spanning both peri and post menopause.

Still, we now see how you and others might wonder at the terminology when looking first at the 2 studies with 35-year old women. So, we have made 3 changes to the manuscript: 1.1, 1.2, captioned above, and 1.3 below. On line 83 we modified the language in the following way:

**1.3 Every study met five inclusion criteria: 1) All the women were prospectively studied during their peri/postmenopausal years and had no invasive breast cancer history at enrollment**

2.

Line 91. Beginning with the statement “unfortunately…” This is unclear what the authors are saying. Please clarify.

The most valid statement is the point that cancer registry calculator using new incidence case not new person with IBC as numerator; I was not aware that was how the numerator was calculate. However, if this was changed to be first IBC what would the risk be? To me adding this piece of information would significantly strengthen the paper.

**………………**

**Response:**

Valid and consistent data are not available because the individual registries receive information provided by clerical staff in hospitals and medical centers. We did evaluate our local region when the 2^nd^ author was the Chief of ObGyn at one of the hospitals in the consortium of 3 and chair of medical education in ob./gyn in the Main Line Health System, near Philadelphia, PA. We wanted to learn how the data are collected and reported to the CDC. And we found that accurate information about prior cases from different hospitals the patient had used was not reported or often even available.

Therefore, to your point “if this was changed to be first IBC what would the risk be?” we really cannot estimate the risk using the retrospective method that the CDC uses because the raw data are not collected, and they are unavailable. However, we do know that because cases are counted instead of women, this forces **the calculated risk estimate** upward because it counts recurrences. Therefore, the actual risk for the first IBC must be lower than the CDC/ACS risk estimates that are using retrospective methods. **The valid** way to accurately calculate this risk, for the first ever IBC, is via prospectively collected data using studies of women who are free of IBC history at the enrollment in the study and who are actively followed up to see the outcome.

We recognize that the CDC does what’s feasible with the data they have available and have worked to find a better way.

We have modified the corresponding text you are referring to on line 94 and expanded it from one to 2 paragraphs to clarify this issue.

3.

Table 1 is a nice outline of the differences. My understanding was the cancer registry used the number of cases, not an estimate of number of cases. Do you mean the current year’s estimate? They always give an estimate for the current years since the case ascertainment has a long lag tail for inclusion. Point 4 is unclear to me. If the registry uses incident IBC, why is follow-up needed. In xx year, this is the number of women with IBC as numerator and denominator in as estimate of number of women who have to possibility of being dx with IBC.

**………………**

**Response:**

We have modified Table 1 to more accurately characterize the way the Retrospective Registry Method contrasts with our new Prospective Method to generate risk estimates for IBC diagnosis. **Please see our revised Table 1.**

4.

Table 2 is a nice outline. However, I would delete the repetitive statement in bold, ‘1st incidence of IBC’ for each study. There was some inconsistencies in description; some used menopausal status, some used study type (screened or not screened), some used HRT, some used menopausal symptom medication. The purpose of this table is unclear since similar items were not included for each study---it lacks consistency. For example, many start with bolded study type (Cohort or RCT, but then others drift such as 11, record review (isn’t that a retrospective cohort study) or #15 was ?? or #20 and #21 did not specify study design. Some use menopausal status and other used age (with some clearly menopausal). No indication of any of these interventions that could protect against IBC. Please address/add info for consistency.

**………………**

**Response:**

We have updated the table to offer the missing consistency; while maintaining accurate descriptions of why each study was undertaken. Our analysis was not designed to compare outcomes of intervention studies. But, where they occurred relevant to the purpose of our investigation, they are described in table 2 and the separate outcomes shown in Table 3. See for example Studies 7 and 8 as well as Study 19.

******We have modified the sentence on line 107-108 of the manuscript you reviewed, and now on lines 114-115, in bold

**4.1 “**Table 2 summarizes the design of 21 studies noting all interventions **and separate outcomes when relevant to IBC diagnosis”.**

******We have made the following improvements in the table

**4.2 We removed the boldface repetitive “1^st^ Incident of IBC”**

**4.3 we inserted missing information**

1. **Study 9 inserted “intending”** before “to show …reduced screening
   i. **inserted** a new sentence

**“Because the outcome did not differ at 14 years follow-up, the data are combined”**

2. **Study 10 inserted “Prospective”** before “Cohort…”
3. **Study 11 Inserted “Retrospective cohort study”:** in place of “Record review…”
4. **Study 12 inserted “contrary to the hypothesis “** before “Baseline estradiol levels did not predict subsequent breast cancer”
5. **Study 12 inserted “cohort”** to now read “**Prospective cohort study of…**
6. **Study 15 Inserted “Retrospective record review of”** in front of current text.
   i. inserted **“at start of this Prospective cohort study of"** physical activity”
   ii. deleting “follow-up”
7. **Study 17 inserted “Prospective”** before “Cohort…”
8. **Study 20 Inserted “cohort”** to now read “**Prospective cohort study of …**
9. **Study 21 Inserted “cohort”** to now read “**Prospective cohort study of …**

We used the defining criteria that each study provided. So, in some cases, that was menopause, others HRT, osteoporosis etc. For example, some studies of “menopausal” women included both those with age ≥50 and those 35 and older who had been ovariectomized (castrated) at hysterectomy. Castration induces immediate menopause because it removes the chief source of estrogen and progesterone production. This is why even 35-year-old women in such studies are correctly categorized as biologically menopausal.

For studies 7 and 8, these 2 RCTs of HRT were not designed to test menopausal symptom alleviation. They were testing CVD risk reduction in high risk populations which is beyond the scope of our investigation. However, these studies do meet our criteria. All 21 studies in Table 2 met our rigorous criteria regardless of what they were designed (and funded) to evaluate.

We recognize that each study was developed for a different purpose; but the stability of the outcome appears to be a strength of our investigation rather than a limitation. These 21 widely diverse studies allow us to form a striking picture as shown in Figures 1 and 2 and 3 and Statistic outcome Tables 4 and 5, of how women with no prior IBC actually fared as they were followed up. We have added this concept to our limitations section.

5.

For the RCTs was only controls used in the analyses? It seems not, and therefore it makes me wonder if the intervention affected IBC. Any way to address this concern?

**………………**

**Response:**

Three RCT trials are presented in table 2 and 3 which show each of the two subgroups for each of the 3 studies. The two different data points for Studies 7,8 and 19 are presented for each subsample. This is why the 21 separate studies produced 24 separate points of data plotted in figure 1. Please see the expanded description of the Sensitivity Analysis for the statistical evaluation and conclusions relevant to the impact on our findings addressing the questions our study was formed to address.

Table 3 is good place to look to see how closely similar each of the subsets were to each other. For the Prempro **trial Women’s Health Initiative** (WHI)I study #7, for example 98.47% of placebo users vs 98.05% of Prempro users were still free of a diagnosis of IBC at 5.2 years. Similar presentations for the WHI hysterectomized sample at 7.1 years that tested Premarin vs placebo can be seen in the table under Study #8. Likewise, Study #19 92.77% vs 93.02% were still free of a first diagnosis of IBC as shown in the table at 21.9 years after enrollment. You can see on Table 2 how that study was designed to test to value of mammogram when protocol trained nurses and doctors did physical exams with and without the radiation adding on screening of mammograms. Their separate conclusions in these 3 RCTs) have each fueled a great deal of controversy in the scientific literature, but these controversies are beyond the scope of this metanalysis.

We have also included, in the limitations section, beginning at line 383 a comment about these different purposes of the study designs, and the coherent picture, nonetheless, that does emerge as we address the question our study was designed to analyze.

6.

The other concern about these cohort studies in which screening was a key aspect AND the outcome for this study was IBC is that we all know screening can pick up in situ BC so this seems to influence the numerator. How was this bias handled?

**………………**

**Response:**

DCIS prior diagnosis was not an exclusion factor. We are only searching for first IBC and whether someone had a prior dx of DCIS was not an impedance to our selection of the 21 studies. The 5 criteria listed in the methods section describe how our studies were selected. As explained in Item 13 of this response DCIS has been shown to elevate the subsequent findings of IBC when it is treated with excision and radiation.

However, if DCIS findings did lead to treatments that then elevated the subsequent findings of a 1st IBC then such a bias would elevate the incidence found. For screened women, this would bias our results towards a larger incidence of IBC (or towards lower freedom) than we actually found. But the incidence we are finding is already low (the freedom we are finding is very high). This is despite, not because, this potential bias. Therefore, in this regard, our results provide a lower boundary for the freedom rate. Freedom would actually be a little bit higher than we found.

Please also refer to Item 13 to see why we believe this important subject is beyond the scope of the present metanalysis but also see item 15 for our expansion of the limitations section that does now include the broader DCIS issue in the manuscript.

7.

Table 2 provides important information. Can you do the age adjusted rate here instead of just crude percent since age is one of the most important variables in cancer dx.

**………………**

**Response:**

Please see where we have addressed this question in points 10 and 11 below

8.

Fig 1, just include 90% to 100% on the y-axis so this can be read for easily. The title of y says percent of women without IBC dx and it is at 100% . It doesn’t make sense that all women or 50% or 70% of women would have IBC at baseline. It looks like it starts at baseline with 0% free IBC and goes to around 7-8% with IBC at 25 years. The description is better than the image and the image can be improved upon.

**………………**

**Response:**

In response to your suggestions to improve the image, we have adjusted the image of Figure 1 in two ways.

**8.1 We have relabeled the x axis to start at 2.6 years which corresponds to the first follow-up outcome.** Since there can be no follow-up at time zero, it does seem best to remove the “0” label which could cause confusion.

**8.2 We have removed the vertical arrow cartoon from the graphic.**

The improved-graph now displays a large white area and visually articulates the huge proportion of women, 99.7%- 93%, who remained free of a diagnosis of IBC.

If the y-axis scale were altered to show only 10% of the range of possible outcomes, then a steep slope would be visually displayed and distort the take home message.  To statistically unsophisticated viewers a steep slope would incorrectly imply that there is a rapid fall off of IBC freedom as the years pass.

The shallow slope in our fully scaled graph most clearly depicts how small the decline was in freedom from a diagnosis of IBC across the 25-year timeline of our studies. The image also shows that the 24 plotted data points are extremely close to this regression line without outliers high above or below the regression line.

Food and Drug Administration regulations meticulously restrict alterations of graphs of clinical investigation results that could mislead physicians and researchers. PLOS One also provides extensive guidelines to avoid inappropriate image manipulation. The authors firmly believe shrinking 10-fold the white space of this graph could impair our communicating the central results of our study.

PLOS One guidelines affirm this principle of “Avoiding inappropriate image manipulation” and the referential paper entitled “What’s in a picture? The temptation of image manipulation” illustrates this phenomenon well, even if they mostly refer to other types of issues and manipulations. The distortion of scale in our figures would fit this principle, as well as the same principles as imposed by the FDA.

9.

From what I could understand from Table 4, everything is significant at <0.05. That is not surprising given the sample size. Given this sample size, it seems reasonable to only consider significance at the <0.001. Overall this table is unreadable. Please use footnotes to help describe all our models. Please add number of participants in each model since it seems this varied. Years means ? PM=1 means; PM=0 means? Overall this is a very unclear table.

**………………**

**Response:**

We agree that this table was difficult to read as it contained a lot of information. We have modified Table 4 for clarity and simplicity. We have expanded our explanation of the statistics involved so that the new table 4 and its 2 supplementary tables will be easier to follow. **Please see the modified Table 4 as well as the 2 supplementary files that are now provided for clarification.** The following is a summary of some of the changes made to the tables:

1) We present in Table 4 only the Generalized Least Squares (GLS) regression results since they are superior as they correct for heteroscedasticity. We moved the OLS results to supplement S4 (that is, supplement 1 of Table 4**)**.
2) We kept only the first five columns instead of all eight columns. The analysis of the extra three columns is now presented in the supplement. See supplement S4.
3) We re-labeled the variable “Years” so that instead of “Years” the table now shows “Effect of each additional year of follow-up”
4) We now clearly labeled our subsamples to be accurate and descriptive, to the best of our abilities, of the important subgroups of studies and characteristics of women analyzed.
5) We now use footnotes explaining each model and how to read the table.

Overall, we believe that the new table and its supplements are clearer and more readable. Thank you for your suggestions.

10.

I’m struggling with the concept of free (probably not use freedom) from IBC dx, that does not take into consideration age of participant.

**………………**

**Response:**

While there is some variation in the data regarding the age of the participants, the variation is minimal and mostly reflect the calendar, i.e. **chronological** age, not the biological age. Since the biological age of the participants is unavailable to us, a deep and meaningful study of age is not feasible at this point and it would be beyond the scope of this research. To verify that the effect of **chronological** age on our findings was insignificant, we performed additional statistical tests controlling for the minimum (calendar) age of the participants at enrollment. The results of these tests are that observed age is not driving the results. In other words, our regression results taking minimum (calendar) age of the participant at enrollment into consideration confirmed that the effect of calendar age on our findings was essentially random statistically and negligible in magnitude from a medical perspective. Please see point 11 below where we expand on the details of the tests.

The implication of these analyses is that, given the inclusion criteria for the studies used in this systematic review and meta-analysis study and particularly the statistical assessments that tested only peri/post-menopausal women without a prior history of IBC, the concept of being free from IBC dx stands regardless of the chronological age of our participants.

In response to our use of the term “Freedom” we have added a final sentence to the paper to explain the existential purpose of the term:

**Our striking finding of freedom from an IBC diagnosis in women who never had one increases optimism and reduces anxiety in their breast cancer expectations. We believe those changed expectations provide a significant societal benefit.**

11.

Another piece of the data that makes me worried is the over-representativeness of the UK Million Women Study. Almost ½ of the participants come from this group and the fu was the shortest at 2.6 years. If the analyses were run without this study, would similar results be reported? I read further that you did an analysis where age and sample size was addressed (lines 217-235). I don’t fully understand the test. How was the independent variables constructed? Number of women enrolled entered into the model as ? Put years studied (means followup period?) and sample size … and found similar result, ‘holding number of women constant’. I just don’t follow the entire paragraph. As far as I can tell this does not address age + followup period as important in the analysis.

**………………**

**Response:**

We re-ran the analysis without the UK Million Women Study and confirmed that the results were unchanged qualitatively. See supplement S5 (that is, the second supplement to **Table 4**).

For a technical description of the specific tests run (including the variables, hypotheses, and how exactly the tests were conducted), we run the following regression models:

$$\boldsymbol{\%free of IBC=}\boldsymbol{\alpha}_{\boldsymbol{0}}\boldsymbol{+}\boldsymbol{\beta}_{\boldsymbol{o}}\left( \boldsymbol{FollowUp Years} \right)\boldsymbol{+}\boldsymbol{\epsilon}_{\boldsymbol{0}}$$

(Model 0 = Baseline Model)

$$\boldsymbol{\%free of IBC=}\boldsymbol{\alpha}_{\boldsymbol{1}}\boldsymbol{+}\boldsymbol{\beta}_{\boldsymbol{1}}\left( \boldsymbol{FollowUp Years} \right)\boldsymbol{+}\boldsymbol{\gamma}_{\boldsymbol{1}}\left( \boldsymbol{MinAge at Enrollment} \right)\boldsymbol{+}\boldsymbol{\epsilon}_{\boldsymbol{1}}$$

(Model 1 = Model Adjusting for Minimum Calendar Age at Enrollment)

$$\boldsymbol{\%free of IBC=}\boldsymbol{\alpha}_{\boldsymbol{2}}\boldsymbol{+}\boldsymbol{\beta}_{\boldsymbol{2}}\left( \boldsymbol{FollowUp Years} \right)\boldsymbol{+}\boldsymbol{\lambda}_{\boldsymbol{2}}\left( \boldsymbol{Sample Size of Study} \right)\boldsymbol{+}\boldsymbol{\epsilon}_{\boldsymbol{1}}$$

(Model 2 = Model Adjusting for the Sample Size of the Study)

The relevant hypothesis tests are **H0:** $\boldsymbol{\gamma}_{\boldsymbol{1}}\boldsymbol{=0}$ **versus H1:** $\boldsymbol{\gamma}_{\boldsymbol{1}}\boldsymbol{\neq0}$ for model 1, and

**H0:** $\boldsymbol{\lambda}_{\boldsymbol{2}}\boldsymbol{=0}$ **versus H1:** $\boldsymbol{\lambda}_{\boldsymbol{2}}\boldsymbol{\neq0}$ for model 2.

The null hypothesis (H0) for the first test (Model 1) is that there is no effect of age on freedom from the first IBC diagnosis. The corresponding alternative hypothesis (H1) is that there is a significant effect of age on freedom from the first IBC diagnosis. We write that, statistically, as **H0:** $\boldsymbol{\gamma}_{\boldsymbol{1}}\boldsymbol{=0}$ **versus H1:** $\boldsymbol{\gamma}_{\boldsymbol{1}}\boldsymbol{\neq0}$**.**

For the second test (Model 2), the null hypothesis (H0) is that there is no effect of sample size on freedom from the first IBC diagnosis. The corresponding alternative hypothesis (H1) is that there is a significant effect of the sample size on the freedom from the first IBC diagnosis. We write that, statistically, as **H0:** $\boldsymbol{\lambda}_{\boldsymbol{2}}\boldsymbol{=0}$ **versus H1:** $\boldsymbol{\lambda}_{\boldsymbol{2}}\boldsymbol{\neq0}$**.**

Those are the relevant hypotheses tests that we are performing. After conducting those tests correcting and not correcting for heteroskedasticity using a student’s t statistical distribution for the estimated model’s coefficients. We performed those tests with and without the UK Million Women Study in our sample. The conclusions of the tests are that we do not reject the null hypothesis in either test, because the results were not significant.  This lack of significant differences suggests that minimal (chronological) age at enrollment and the sample size of the study **do not significantly affect** the regression and that no bias is incurred in the main findings associated with those variables.

Because of the scientific nature of the tests we are performing, we “do not reject” the null hypothesis instead of “accepting” it. The null hypothesis is assumed true until proved false, so we cannot accept it, but rather "not reject it". The same scientific rationale applies to theories and even in judicial trials. In a trial, innocence should be assumed unless proven guilty. So, the judge decisions are either "guilty" or "not guilty", but never that the court proved someone innocent. This is because, in principle, they assumed (or at least should have assumed) the accused person to be innocent.

See the paragraphs beginning at line 298 of the revised manuscript for our best attempt to describe these statistical tests and insight in words, in an effort to make it simple and concise for the reader, while trying to maintain the style of the paper and the flow of the main ideas.

12.

Probably would not use the word ‘bad health habits’ since this is a judgmental word. Line 250 The strongest argument for this paper is that the conventional use of a numerator for determining bc risk may over-inflate the risk since recurrence is well known. Without knowing what the rate would be by changing the numerator using cancer registry data is a significant limitation in the argument made in this paper.

**………………**

**Response:**

Yes, we agree about removing “bad health habits. Please see

**12.1 line 335 now is changed from**

Some studies even selected for bad health habits, like the WHI (Table 2, studies 7 and 8) with its obese and overweight populations on statins.

**To now say**

Some studies included women with compromised health conditions, like…

We agree with you that it is important to emphasize the strongest argument for this paper in “that the conventional use of a numerator for determining bc risk may over-inflate the risk since recurrence is well known.” We do emphasize this now, throughout the ms, particularly in Table 1, and in the discussion beginning with line 347: “We have previously detailed… and again in the final sentences of the Conclusion

In response to “Without knowing what the rate would be by changing the numerator using cancer registry data is a significant limitation in the argument made in this paper.” These data are not available to us. Therefore, we take a step back from the retrospective methods and take a different, prospective, approach. We believe that a careful follow-up of the subjects is the only way feasible to accurately calculate the incidence (or freedom) **of the first IBC diagnosis.** This is precisely the direction we have taken in this paper.

13.

I also believe the ACS considers both IBC and in situ. About 12-15% of bc are insitu with strong mammogram uptake and this should be noted as part of their calculation. This also reflects a limitation in this analysis, women who were dx with in situ through use of mammograms, never developed IBC and were not ‘counted’ in the statistics. It seems a bit like comparing apples and oranges.

**………………**

**Response:**

We checked, and our citation “Similarly, in 2019, the American Cancer Society posted this statement: “As reported in previous years, a female’s probability of developing invasive breast cancer in her lifetime continues to be 12.4 percent or 1 in 8 women.” is a direct quote. Also, the ACS 2019 data table 6 “Probability of Developing INVASIVE Cancer…” is linked here <https://www.cancer.org/content/dam/cancer-org/research/cancer-facts-and-statistics/annual-cancer-facts-and-figures/2019/probability-of-developing-invasive-cancer-during-selected-age-intervals-by-sex-us-2013-2015.pdf>

It provides the same 1 in 8 lifetime probability calculation for IBC. So, this separate table refers only to invasive cancers and in situ cases are not included. As you commented, there is the obvious problem of ignoring the IBC recurrences thereby leading to multiple case counts incorrectly elevating the risk estimate.

We agree that strong mammogram uptake has also yielded another finding: i.e. that 12 to 15% of breast “pathologies” identified are DCIS. Our studies of IBC incidence did not exclude women with a prior finding of DCIS. So, our data presentations are not affected by the presence or absence of DCIS. The discovery of DCIS during the follow up period in the studies we cited would likely have led to treatment for the DCIS as the women continued to be followed for IBC. This may actually increase the likelihood of a first IBC in our data.

Although the subtleties of DCIS research are beyond the scope of our paper, it is useful to appreciate that DCIS does not prevent subsequent IBC. The impact of DCIS treatments on later incidence of IBC, was reported in 2011

**[https://www.ncbi.nlm.nih.gov/pmc/articles/PMC3107729](https://www.ncbi.nlm.nih.gov/pmc/articles/PMC3107729/) Major Outcomes* of the NSABP B-24 Trial: DCIS RCT after Lumpectomy and Radiation: 1804 women, 5 yrs placebo vs. Tamoxifen 20 mgs/day. They reported that 4.8% of women prescribed 5 years of tamoxifen vs. 8.2% of those on placebo were diagnosed with IBC by 84 months after radiation and surgery. This outcome study also showed that the adverse effects on deep venous thrombosis, stroke, pulmonary embolism and uterine sarcoma of the DCIS treatments may be greater than any possible benefit. Similarly, the increased risk of major coronary adverse effects from the radiation to “treat” breast cancer have also created a great deal of controversy about the wisdom of current treatment protocols for DCIS.

See Darby et al

[Darby](https://www.ncbi.nlm.nih.gov/pubmed/?term=Darby%20SC%5bAuthor%5d&cauthor=true&cauthor_uid=23484825) SC et al, (2013) N Engl J Med. 2013 Mar 14;368(11):987-98. doi: 10.1056/NEJMoa1209825.

Still, we agree that others might also wonder as you did, and now think it relevant to include the topic on the limitation imposed by such risk of IBC after treatment for DCIS. See our answer to item 15 below.

14.

Please provide some reference to support the statement on line 268 that “census estimates are skewed towards undercounting.” Using one example in which an error was made and acknowledged as an error is not sufficient to make this global statement. More documentation of this is needed.

**………………**

**Response:**

**14.1**

We agree that more references would be important and have added 3

additional references describing census undercounts

Kissam E Differential undercount of Mexican immigrant families in the U.S. Census ... Statistical Journal of the IAOS 33 (2017) 797–816 797 DOI 10.3233/SJI-1

<http://www.wkfamilyfund.org/docs/sji-33-sji170388.pdf>

O’Hare W.P. Who Is Missing? Undercounts and Omissions in the U.S. Census. In: Differential Undercounts in the U.S. Census. (2019) Springer Briefs in Population Studies. Springer, Cham

<https://link.springer.com/chapter/10.1007/978-3-030-10973-8_1>

Williams J The 2010 Decennial Census: Background and. Issues. Jennifer D. Williams. Specialist in American National Government. February 3, 2011. Congressional Research Service

https://www.census.gov/history/pdf/2010-background-crs.pdf

15.

The limitation section needs to include a few of the comments made in this review. Many of the known risk factors may or may not have been included in the studies, such as family history of bc.

**………………**

**Response:**

We have inserted the following items in the limitations section of the ms

**15.1,2,3 and 4**

1. DCIS has been shown to elevate the subsequent findings of IBC. However, if DCIS findings did elevate the subsequent findings of a 1^st^ IBC then such a bias would elevate the 1^st^ IBC incidence found. For screened women, this would bias our results towards a larger incidence of IBC (or towards lower freedom). But the incidence we are finding is already low (the freedom we are finding is very high). This is despite, not because, this potential bias. Therefore, in this regard, our results provide a lower bound for the freedom rate.

This limitation then is that Freedom would actually be a little bit higher than we could identify in the mammogram screened samples.

2. We recognize that each of the 21 studies was developed for a different purpose; but the stability of the outcome appears to be a strength of our investigation rather than a limitation. These 21 widely diverse studies allow us to form a striking picture as shown in Figures 1 and 2 and 3 and Statistic outcome Tables 4 and 5, of how women with no prior IBC actually fared as they were followed up.

3. The 21 studies did not systematically test their cohorts for IBC outcome as it related to some of the known risk factors for invasive breast cancer: high alcohol consumption, obesity, certain HRT regimens that include synthetic progestins rather than those regimens that use progesterone, inadequate daily exercise habits, deficient Vitamin D3, and cumulative total radiation doses to the breasts. While these are each recognized it remains for future researchers to examine whether behavioral change can reduce the incidence. Case control trials would be helpful but difficult to manage. See citation to: Cutler WB. Chapter 8, Protecting the Breasts in *Hormones and Your Health: The Smart Woman’s Guide to Hormonal and Alternative Therapies for Menopause.*, Wiley, NY, 2009

4. Data on family history of breast cancer were not provided in any consistent way and, to the degree that women were more likely to be enrolled in a screening program because they are currently cancer free and concerned about their own risk, our findings would be biased as higher than would occur in women without a family history. Likewise, history of a genetic predisposition would also yield the same limitation.

5. Data on the biological age of the participants, especially those surgically menopausal, was unobservable and therefore unavailable to us. A rigorous study of the effects of biological age would be desirable but it is beyond the scope of this research. Nevertheless, the stability of our results across different subsamples of women suggests that variations in biological age may have, if any, a minimal effect on our findings. Also, using calendar, i.e. chronological, age as a proxy for biological age we observed an insignificant and negligible effect of age on the annual decline in the percentage of women who remained free from their first IBC diagnosis. This suggests that even though this limitation is worth exploring in future research, it is unlikely that the main findings will be altered.

16.

Adding information about the bc risk factors (prevalences) by study would be a great addition and likely readily available. This could strengthen the analysis since it represent the population or does not. I would suspect it doesn’t represent lower educational attainment, or minorities (for US).

**………………**

**Response:**

We agree. Please see the limitations we have added as described in item 15 above, and now in the ms.

17.

If the study descriptions are not truly representative this should be included in the limitations and if it does, then that is a strength that should be noted.

**………………**

**Response:**

Please see above. Again, we agree with you and have worked into the limitations section of the ms what we are able to conclude from the present metanalysis.

18.

Overall, I think this is a great analysis and just needs to be tighten up to provide a very strong message.

**………………**

**Response:**

Thank you. We did our best for a rigorous analysis with the information available and we believe that we provide a strong message that will improve the well being of women, especially those without a prior history of IBC. We really appreciate your comments, after which we believe we have a better paper.
