# Supplementary material for: Long term absence of invasive breast cancer diagnosis in 2,402,672 pre and postmenopausal women: A systematic review and meta-analysis
Source: PLoS One. 2020 Sep 10;15(9):e0237925. doi: 10.1371/journal.pone.0237925 (PMC7482842; doi:10.1371/journal.pone.0237925)
Supplement: Attachment — Submitted filename: Response to Reviewer r2.docx [file pone.0237925.s008.docx]

Response from Reviewer 7/10/20

6. Review Comments to the Author

Please use the space provided to explain your answers to the questions above. You may also include additional comments for the author, including concerns about dual publication, research ethics, or publication ethics. (Please upload your review as an attachment if it exceeds 20,000 characters)

Reviewer #1: Thanks for the extensive responses.

1] I appreciate the response to the issue of using peri-menopausal. I am puzzled by the insistence on using this term, including in the title when it does not capture the characteristics of the women and muddles the picture. As mentioned by the reviewers “For the 3 studies that enrolled women who were at least 40:#15,18, and 19 we also looked closely at each before qualifying them as studying women in their peri/postmenopausal era, since the term “perimenopausal” is a loose one – but does convey our intent.” No idea what the authors mean by ‘the intent’. I don’t get it since these 3 studies start at women who are 40 years old. We can argue about this, but ultimately you can choose to use this ‘loose term’ extensively and in the title but be aware that if I read this, I would question your work. My thinking would be, ‘if they ‘misclassify’ women by assigning them a phase of menopausal status that they are not definitely experiencing, then what else were the authors less precise about. At minimum this should be defined in the first paragraph of the paper—perimenopausal is defined in this paper as any women aged 40 and older who has not been classified as menopausal.

**Response 1**: We agree with you and have changed “peri” to “pre” throughout the entire manuscript.

We confirmed that standard usage of the 2 terms renders this change essential.

- [Premenopausal | **Definition of Premenopausal by Merriam** ...](https://www.merriam-webster.com/dictionary/premenopausal)
- [https://www.merriam-webster.com/dictionary/premenopausal**Premenopausal**](https://www.merriam-webster.com/dictionary/premenopausalPremenopausal) **definition** is - of, relating to, or being in the period preceding **menopause**. ... [https://www.**merriam**-webster.com/**dictionary**/**premenopausal**](https://www.merriam-webster.com/dictionary/premenopausal).
- [Perimenopause | **Definition of Perimenopause by Merriam** ...](https://www.merriam-webster.com/dictionary/perimenopause)
- [https://www.merriam-webster.com/dictionary/perimenopause**Perimenopause**](https://www.merriam-webster.com/dictionary/perimenopausePerimenopause) **definition** is - the period around the onset of **menopause** that is often marked by various physical signs (such as hot flashes and menstrual ...

Thank you for reemphasizing this point.
……………………………

2] In response to the request about calculating rate using only first case, the rationale to not do this is not compelling. Of course, at any hospital level, your statement (“And we found that accurate information about prior cases from different hospitals the patient had used was not reported or often even available.”) is not a surprising finding. We are all aware of the fragmentation of health care in the US. Not sure about the other counties.

“Therefore, to your point “if this was changed to be first IBC what would the risk be?” we really cannot estimate the risk using the retrospective method that the CDC uses because the raw data are not collected, and they are unavailable.” This is a puzzling statement. What do you mean ‘raw data are not collected.’ It is a federal mandate to report cancer for any patient and in requesting data from the registry, first dx is a field that must be specified. I don’t understand your rationale for gold standard; using a registry that captures all cases is a much better method to estimate risk, if the numerator excludes those who have a recurrence. I thought this was one of your major points. If not, then I am truly confused. Prospective studies, unless very large and representative of the population AND have incredibly high retention rates (not happening in this era of research) are not the gold standard to determine risk. This paragraph puts a spanner in the paper, to me. Why would you not use cancer registry data but have it set up, in the future, to do a ‘better job’ of calculating risk? Or do you think it is impossible to use cancer registry data for estimating risk? What am I missing since this paragraph suggests that we should basically throw out cancer registry data for estimating risk. Is this really your conclusion?

**Response 2:**

Yes, you are correct that this is one of our major points. And I now SEE what you mean about **registry data. W**e agree that for those registries that can define the number of 1st IBC cases, and the population size in question, and where the resident population is stable it would be doable. Researchers should be able to analyze their results to yield the  incidence of 1st cases at each group over 25 years. We believe this would most likely render very similar outcomes to what we have identified in these 21 studies of 24 sets of data, i.e., outcomes.

It seems that you are referring to health care systems that are more homogenous than those in the US. And you are referring to geographic regions that have none of the worldwide migration we see happening now, with numbers of undocumented persons uncountable all over the world. In such regions, cancer will get recorded if they involve connecting the woman with the health care system. But the number of undocumented persons in the region who do not have a cancer diagnosis, the denominator of the incidence fraction, will be unknown.

In places where the 'universal' health care systems function with rigorously managed registries of all the women in the population such analysis should be possible. Such registry systems are so far ahead of the fragmented health care in US. The CDC only “catches and records cases” but never registers women when they are well to follow up what happens to them.

Because of variation in data collection methods across countries, we worked to find a prospective method to analyze all studies that enrolled women with no prior IBC, that searched for IBC for a given duration and then reported their rigorous findings. We selected only (and all) those 21 published studies that shared the common features listed in our methods section.  We agree with you that some of these 21 studies worked with a usable central registry. But we find others did not. For example, studies 7,8,11, 14 enrolled IBC free women and followed their outcomes independently of registries.

Now that we understand what you were suggesting, we have inserted this paragraph, preceding the final paragraph in the Discussion Section

**Future corroboration of these findings using registry data**, **when available, would be valuable. Data from those registries that can specify the number of first IBC cases and the population size should enable calculation of cumulative total incidence or absence over a given span of years of age, by totaling the incidence in each age group. The expectation is that such alternative analysis would yield outcomes similar to those obtained in the current analysis of these 21 studies of 24 outcomes.**

**………………….**

3] In Table 1, #1: add the denominator is xxx. And for retrospective cancer registry (“estimated number of eligible women in the census for the ….” The key point here is the number of eligible women are estimated. Add ‘eligible’ women to prospective. To me that is the different in #1. One is exact number of eligible women and the other is an estimate of eligible for the denominator.

**Response 3:** We have made these changes.

………………….
4] #2 implies a different way in which women’s bc dx was determined but some/many/most of the studies use the same system. I didn’t read each study, but I would be very surprised if the very large prospective studies did not use cancer registries as linkage to determine dx. Is this incorrect? I just randomly picked one study (Finnish) and they used the registry for followup bc dx.
I want to reiterate that this is a GREAT study and work. I just want to support the representation of the data described to be precise and without question so that it gets the attention it needs vs distracting comments that take away from the message. The only difference between the 2 systems is one uses a list and matches it to the registry and the other just uses the registry list.

**Response 4:** In the current retrospective method employed by the CDC in the U.S., “participants” (the whole population) are not followed-up in the precise way we are able to achieve using our prospective method. No women are enrolled by the CDC risk estimating method. Only cancer cases are counted into the system. In the USA retrospective methods, the risk calculations do not use individual data directly. In turn, periods of data collection are proxies for the “follow-up” period. Therefore, the cumulative incidence of IBC calculated from the CDC retrospective method can only be seen as an approximation using aggregated data.  And even if they could identify only first cases, they would still have the problem of not knowing the population size, i.e., the true denominator of the incidence fraction.

5] #3 This is the key point of the study, to me.

**Response 5:** Yes it is one of the key points
……………..

6] #4 I’m not sure what you mean here. The registries enter date of dx. Longitudinal studies enter date of dx. Longitudinal studies somewhat arbitrarily enroll women on a certain date. Why is that meaningful for this analysis. By default, cancer registries ‘follow’ all women from year of eligibility to date of dx since it is a registry. Cumulative incidence is the number of new events or cases of disease divided by the total number of individuals in the population at risk for a specific time interval. This again gets back to the basic question as to whether the cancer registries can differentiate between first and subsequent cancer dx. I just don’t get this one as a difference between the 2 systems in a meaningful way.

**Response 6:** see above “Response 2 and Response 4”
…………………..

7]#5 is kind of redundant. The ‘known follow-up period’ isn’t useful info for calculating incidence rate. This is using #1 and #3

**Response 7:**  We prefer, however, to leave it even if it is redundant. It would help the reader put #1 and #3 together to compare side by side the calculation of incidence under each method.  

………………………

8] Table 4. What is observation? Is this cancer dx? Besides this question the table is so much easier to understand. Thanks

**Response 8] No.** We have changed the word ”observation” throughout the ms to “outcome” and inserted a missing “N” in front of that word in the table. For example, in column 1, the Full Sample, the reader of the table now sees a consistent description. The N equals the Number. For example, in column 1, covering all the studies: There were an ‘N’ of 24 separate outcomes in the 21 studies of 2,402,672 women. The outcomes are the data points that are shown in each figure and in Table 3 : one data point for each study or study subgroup. Thank you for pointing out the confusion since we had been missing the “N’ and we do not want to make the reader guess! So, inserting the missing “N” in front makes it more consistent for the reader of the table to decipher.

…………………………

**Table 4 – Sensitivity analysis**

*Dependent Variable: Percentage of women free of invasive breast cancer (IBC), the “survival” rate, at the end of each study.*

|  | (1) | (2) | (3) | (4) | (5) |
| --- | --- | --- | --- | --- | --- |
|  | Full  sample  of all studies | Subsample  of studies limited to post-menopausal women | Subsample  of studies not limited to menopausal women | Subsample  of studies limited to  screened  women | Subsample  of studies not limited to screened women |
| Effect of each | **-0.255^***^** | -0.228^***^ | -0.254^***^ | -0.256^***^ | -0.214^**^ |
| additional year of follow-up | **(0.0000)** | (0.0000) | (0.0005) | (0.0000) | (0.0026) |
| Constant | 99.78^***^ | 99.71^***^ | 99.61^***^ | 99.79^***^ | 99.52^***^ |
|  | (0.0000) | (0.0000) | (0.0000) | (0.0000) | (0.0000) |
| N (Outcomes) | 24 | 16 | 8 | 17 | 7 |
| N (Studies) | 21 | 14 | 7 | 14 | 7 |
| N (Women) | 2,402,672 | 1,808,022 | 594,650 | 2,264,591 | 138,081 |
| R-squared | 0.920 | 0.984 | 0.882 | 0.921 | 0.861 |

Statistical *p*-values are shown in parentheses (^**^ *p* < 0.01, ^***^ *p* < 0.001). The generalized least squares (GLS) regression method was used to correct for heteroskedasticity stemming from unequal error variances across the disparate studies.

……………………

9] I disagree with the final sentence since the finding of this great analysis was not about the patient’s anxiety and optimism but rather the risk of breast cancer diagnosis by the general population. One could easily argue that this finding can have the opposite effect on the general population of reducing compliance with breast cancer screening since it looks like the risk is much lower than publicly portrayed, from these analyses. If these findings are accepted as norm, we could reasonably go back to the 1980s rates of screening and subsequent more late stage cancer dx. I would not call that scenario a significant societal benefit. I’m not convinced that low risk women (such as no family hx) are anxious about being diagnosed with breast cancer. To me this last sentence distracts from the findings by injecting a personal perspective that could easily be countered.

I also note that this nuance, non-neutral word ‘freedom’ is also used in table footnote. I already indicated my perspective that using this word choice may do a disservice to this work due to inserting non-neutral language.

**Response 9:** We agree that the purpose of this paper will be better served by maintaining neutral language as much as possible. We have therefore deleted the last 2 sentences. And we have altered the non-neutral “freedom” term as shown in the title and throughout the ms. We replaced “freedom from” with “absence of” and several other similar variations.

…………………

10] I would appreciate a definition with maybe an example to understand chronological age vs participant’s age, in the paper.
**Response 10]**

**The chronological age is the participants’ age in this paper. The biologic age of the participants is unknown.**

**We have altered the paragraph in limitations to add SEVERAL SENTENCES with references. It now says:**

Chronological age calculates the age in years from the date of birth.  Biological age reflects the actual physiologic state: whether one is aging faster or slower than the chronologic age would imply. Data on the biological age of the participants, especially those rendered surgically menopausal, were unobservable and therefore unavailable to us.

Castration (ovariectomy) ages a 35 to 45-year-old woman into immediate menopause, making her biologically older than her intact peers. And even hysterectomy, with preservation of the ovaries, accelerates aging. For example, it triggers a demonstrable acceleration of kyphosis and related loss of bone^[1]^.

………………………

11] Appreciate seeing the formulas for the analyses since that was much easier to follow than the description as well as a more information in the limitation section

**Response 11]** Thank you for all your comments and suggestions. We think they helped make a better paper and are grateful.

1. Cutler W Chapter 8 Protect Your Bones - with Exercise, Posture, Vitamin D, Calcium, and Hormones. , in . **Hormones and Your Health: The Smart Woman's Guide to Hormonal and Alternative Therapies for Menopause.** © 2009 John Wiley & Sons, Inc.

   Cutler WB, Genovese-Stone E  Wellness in Women After 40 Years of Age: The Role of Sex Hormones and Pheromones: Part II Hormone Replacement Therapy; Part III Hysterectomy. **Current Problems in Obstetrics, Gynecology and Fertility** 2000; 23:33-88.

   Cutler WB, Karp J, Stine R (1988) Single photon absorptiometry imaging as a screen for diminished dual photon density measures. **Maturitas** 10:143-55.

   Sincerely,

   Winnifred Cutler, PhD

   Corresponding Author
